# Treatment of Autoimmune Bullous Diseases During Pregnancy and Lactation: A Review Focusing on Pemphigus and Pemphigoid Gestationis

**DOI:** 10.3389/fphar.2020.583354

**Published:** 2020-10-02

**Authors:** Giovanni Genovese, Federica Derlino, Emilio Berti, Angelo Valerio Marzano

**Affiliations:** ^1^Dermatology Unit, Fondazione IRCCS Ca’ Granda Ospedale Maggiore Policlinico, Milan, Italy; ^2^Department of Pathophysiology and Transplantation, Università degli Studi di Milano, Milan, Italy; ^3^Dermatology Unit, ASST Santi Paolo e Carlo, Milan, Italy

**Keywords:** autoimmune bullous diseases, pemphigus, pregnancy, lactation, management, treatment

## Abstract

Pregnancy may induce the onset or exacerbation of autoimmune bullous diseases such as pemphigus or pemphigoid gestationis. A shift toward T helper (Th) 2 immune response and the influence of hormonal changes have been evoked as possible triggering factors. Therapeutic management of this setting of patients may represent a challenge, mainly due to safety concerns of some immunosuppressive drugs during pregnancy and lactation. In this narrative review, we provided a comprehensive overview of the therapeutic management of autoimmune bullous diseases in pregnant and breastfeeding women, focusing on pemphigus and pemphigoid gestationis.

## Introduction

Autoimmune bullous diseases (AIBDs) represent a group of mucocutaneous disorders that encompass different conditions hallmarked by autoreactive antibodies directed against epithelial adhesion molecules (Egami et al., 2020). Based on the localization of the blister, AIBDs are distinguished into two different categories: (i) AIBDs with intraepithelial cleavage, which belong to the pemphigus group (Kridin, 2018), including pemphigus vulgaris (PV) and pemphigus foliaceus (PF), and (ii) AIBDs with subepithelial detachment, which comprise diseases of the pemphigoid group (Amber et al., 2018) such as bullous pemphigoid (BP), pemphigoid gestationis (PG), mucous membrane pemphigoid (MMP), linear IgA bullous dermatosis (LABD), and epidermolysis bullosa acquisita (EBA). Among all these entities, PG and PV are those which involve more commonly women of childbearing potential (Feliciani et al., 2019). In particular, PG is regarded as a genuine specific dermatosis of pregnancy (Beard and Millington, 2012). On the other hand, PV and PF tend to occur frequently in young females and pregnancy may induce the onset or exacerbation of these diseases (Zhao and Murrell, 2015). Indeed, a shift toward T helper (Th) 2 immune response and the influence of hormonal changes have been indicated as possible triggering factors of AIBDs during pregnancy (Feliciani et al., 2019). Given that neonatal health is closely linked to the control of the underlying maternal AIBD, the issues related to the management of these disorders during pregnancy and lactation take on remarkable importance, often requiring a close interaction among dermatologists, obstetricians and pediatricians. Furthermore, the unsafety during pregnancy or breastfeeding period of several immunosuppressive agents used to treat AIBD poses a major therapeutic challenge in this peculiar setting of patients (Kushner et al., 2018). On the other hand, achieving disease control can be hindered by pregnancy-associated hormonal changes and pregnancy complications (pre-eclampsia, gestational diabetes, intrauterine growth restrictions, and preterm birth). Both in pemphigus and PG, maternal autoantibodies can be transferred to the newborn determining the occurrence of neonatal blistering lesions. Neonatal disease usually resolves spontaneously within 1–4 weeks and, only in rare cases with widespread blisters, oral corticosteroids can be used to control the disease (Tavakolpour et al., 2017). Finally, randomized controlled trials on therapeutic options for AIBD in pregnancy are lacking due to the impossibility to perform them in this subset of patients.

In this narrative review, we have striven to provide a comprehensive overview of the management of AIBDs during pregnancy and lactation, focusing on pemphigus and PG, merging our experience with data extrapolated by case series and reviews.

## Treatment of Pemphigus During Pregnancy

**Figure 1:**
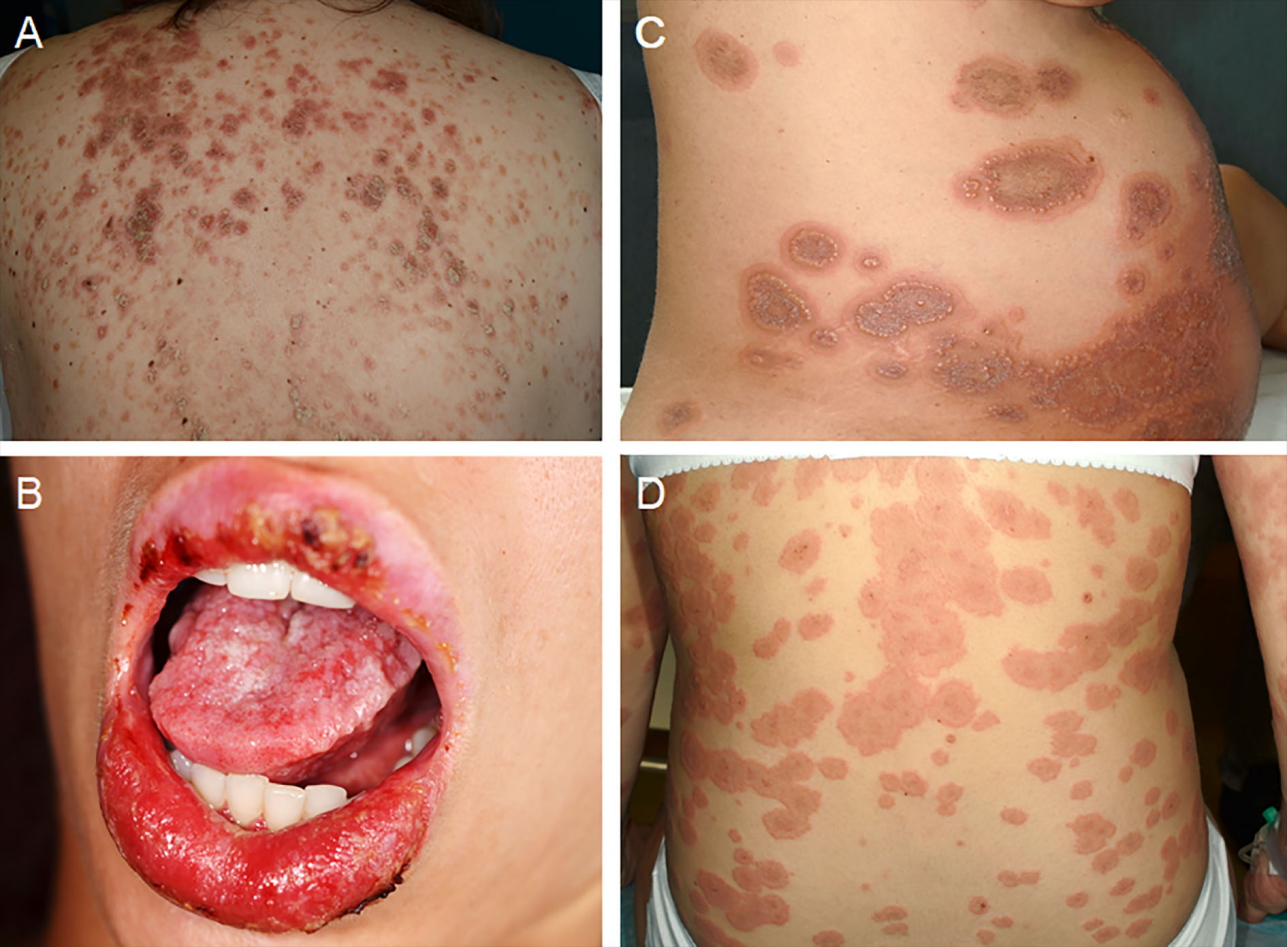
Clinical features of autoimmune bullous diseases during pregnancy. **(A)** Erythematous-scaling lesions on the back of a pregnant patient with pemphigus foliaceus. **(B)** Oral erosions in a pregnant patient with pemphigus vulgaris. **(C, D)** Urticarial plaques associated with vesiculobullous lesions in two pregnant patients with pemphigoid gestationis. Written informed consent was obtained from all patients for the publication of clinical images.

**Figure 2:**
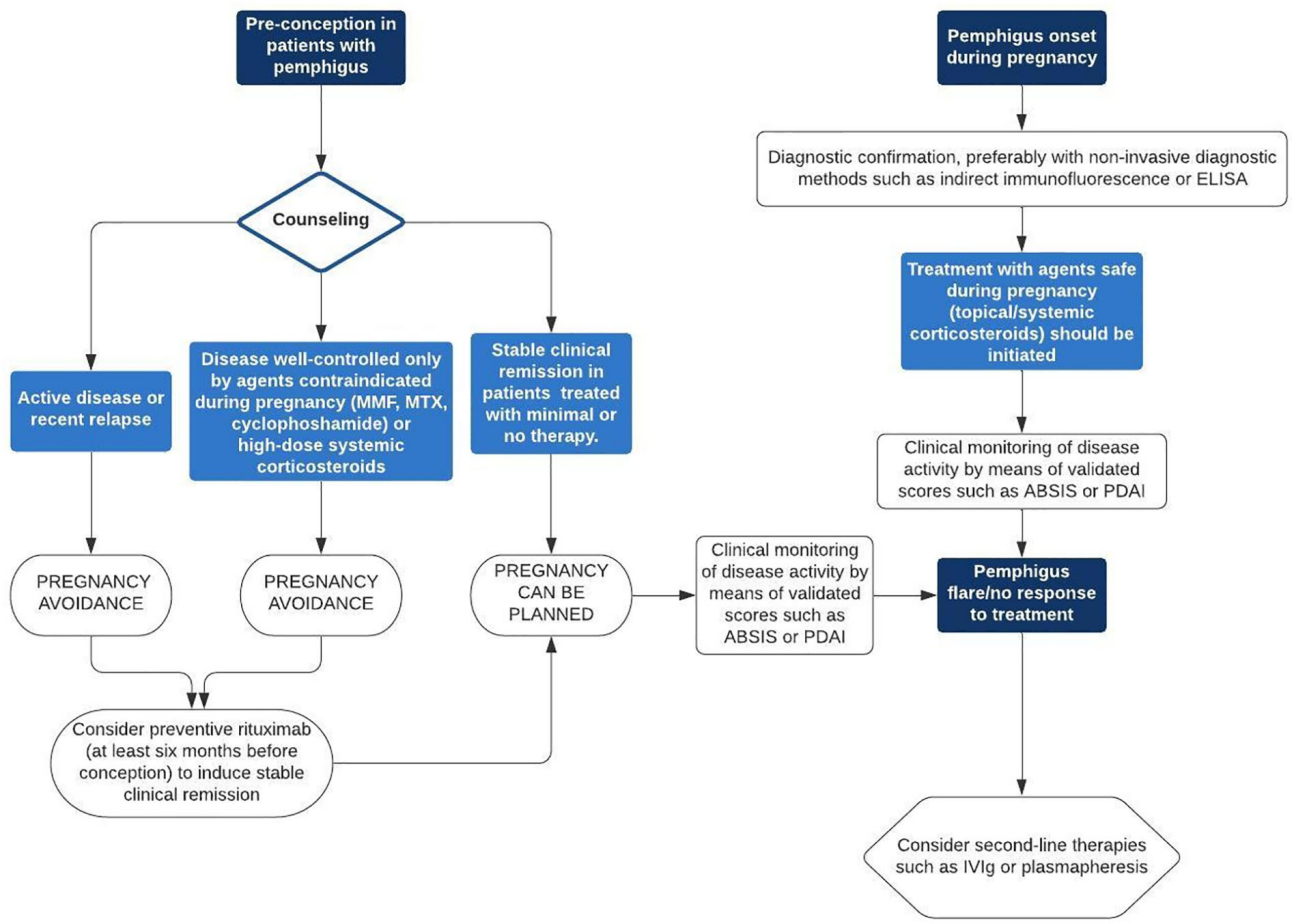
Management algorithm for patients with pemphigus who plan conception or pregnant women who experience pemphigus onset, flare or poor response to treatment. ABSIS, Autoimmune Bullous Skin Disorder Intensity Score; ELISA, enzyme-linked immunosorbent assay; IVIg, intravenous immunoglobulin therapy; MMF, mycophenolate mofetil; MTX, methotrexate; PDAI, Pemphigus Disease Area Index.

Both PV and PF may appear for the first time (Kokolios et al., 2017) or relapse (Green and Maize, 1982) during pregnancy, showing the same clinical features observed in other subsets of patients (**Figures 1A, B**). In both cases, pregnancy needs to be classified as “high-risk” and the severity of the maternal disease requires to be controlled with safe agents and close monitoring of both mother and fetus. The same applies for patients who plan breastfeeding (Kardos et al., 2009). Women of childbearing age affected by pemphigus should be advised to avoid conception if they are on therapy with methotrexate, cyclophosphamide or mycophenolate mofetil. If on treatment with these drugs, they must be stopped and replaced. It is worth noting that pemphigus treatment in pregnant women is aimed at preventing both neonatal pemphigus (Zhao et al., 2016) and adverse pregnancy outcome such as preterm birth, low birth weight pre-eclampsia, stillbirth, and spontaneous abortion (Kardos et al., 2009). **Figure 2** shows a management algorithm based on current evidence (Kushner et al., 2018) and our experience for patients with pemphigus who plan conception or pregnant women who experience pemphigus onset, flare or poor response to treatment. Immunosuppressive treatments commonly used to treat pemphigus such as mycophenolate mofetil (Food and Drug Administration [FDA] pregnancy category D), cyclophosphamide (FDA pregnancy category D), and methotrexate (FDA pregnancy category X) have been contraindicated during pregnancy by the most representative Societies of Rheumatology (Flint et al., 2016; Götestam Skorpen et al., 2016; Sammaritano et al., 2020) and Gynecology (Committee on Obstetric Practice Society for Maternal-Fetal Medicine, 2019). The use of tetracyclines, which have been reported to be effective in some cases of pemphigus (Hone and Mutasim, 2016), is also discouraged by the FDA due to their teratogenicity and bone growth disruption (Kushner et al., 2018). Data on azathioprine (FDA pregnancy category D) safety during pregnancy are controversial (Patsatsi et al., 2019). Even if it some initial studies reported increased risk of congenital defects in babies born from mother treated with azathioprine (Nørgård et al., 2003), more recent evidence seem to suggest that no association between fetal exposure to azathioprine during pregnancy and birth defects (Goldstein et al., 2007) or other adverse pregnancy outcomes such as low birth weight (Akbari et al., 2013) exist. The 2020 guidelines and recommendations endorsed by the American College of Rheumatology recommend azathioprine as compatible for use throughout pregnancy (Sammaritano et al., 2020).

Women treated with mycophenolate mofetil, methotrexate or cyclophosphamide should be advised to discontinue therapy 6 weeks, 3 months and one ovulation cycle before conception, respectively (Patsatsi et al., 2019). The authors of a very recent systematic review on the effect of paternal exposure to immunosuppressive drugs on fertility, pregnancy and offspring outcomes concluded that data regarding pregnancy and offspring outcomes were limited but no significant negative effects associated with paternal immunosuppressant exposure were reported (Perez-Garcia et al., 2020).

## Treatment of Pemphigoid Gestationis

PG is a subepithelial autoimmune blistering disease associated with pregnancy, puerperium, or, more rarely, gestational trophoblastic disease (**Figures 1C, D**) (Ambros-Rudolph et al., 2006). Although it usually resolves after delivery, it may also show a persistent or chronic-relapsing course, with flares usually occurring after delivery, during menses, or in association with the use of hormonal contraceptives. In cases associated with gestational trophoblastic disease, the treatment of underlying condition may facilitate PG remission (Slazinski and Degefu, 1982; do Valle Chiossi et al., 2000; Djahansouzi et al., 2003). The treatment is non-standardized, with most evidence derived from individual case reports or small series, currently there being no specific guidelines. In our experience, high-potency topical corticosteroids as monotherapy can be used in mild cases, while systemic corticosteroids (prednisone 0.5 mg/kg/day) should be reserved for moderate-to-severe cases. In case of inadequate response, it is reasonable to increase the dosage of systemic corticosteroids or consider adding/replacing systemic corticosteroids with a steroid-sparing agent (e.g., dapsone or intravenous immunoglobulin therapy [IVIg]).

## Therapeutic Options for Pemphigus and Pemphigoid Gestationis During Pregnancy

### Systemic Corticosteroids (FDA Pregnancy Category C)

In pregnant patients with pemphigus, systemic corticosteroids alone or in combination with topical corticosteroids or steroid-sparing agents such as azathioprine or IVIg, are the mainstay of treatment, as suggested also by the case series of Daneshpazooh et al. (2011) and in the reviews by Lin et al. (2015) and Kardos et al. (2009). The good safety profile during pregnancy and lactation and the effectiveness of these agents make low-dosage (less than 20 mg/day) (Murase et al., 2014) systemic corticosteroids a first-line therapeutic option in this setting. Systemic corticosteroids, sometimes combined with topical corticosteroids, seem to be the mainstay also for PG treatment, particularly during pregnancy and in mild-to-moderate cases (Piva et al., 2014; Veiga et al., 2018).

### Topical Corticosteroids (FDA Pregnancy Category C)

Topical corticosteroids may represent a first-line treatment in patients with stable pemphigus, either as monotherapy (Moncada et al., 1982; Chowdhury and Natarajan, 1998; Çayırlı et al., 2015) in mild disease or in combination with systemic corticosteroids in more severe cases (Hern et al., 1998). Although they have been demonstrated to be not associated with severe adverse pregnancy outcome (Chi et al., 2016), very potent topical corticosteroids may cause fetal growth restrictions. This is due to a systemic absorption, most encountered when used on the skin of eyelids, genitals and folds. Successful treatment with topical corticosteroids as monotherapy has also been reported in PG, particularly for mild or localized skin lesions during pregnancy but also after delivery, for mild relapses (e.g., during menses) (Cozzani et al., 2005; Fukuda et al., 2012; Ingen-Housz-Oro et al., 2016).

### Topical Calcineurin Inhibitors (FDA Pregnancy Category C)

Topical tacrolimus and pimecrolimus are poorly absorbed systemically because their molecular size prevents skin penetration. For this reason, although there are no controlled trials on topical calcineurin inhibitors in pregnant women with AIBDs, it has been suggested that, when no alternatives exist, their use on small areas is admissible (Murase et al., 2014).

### Dapsone (FDA Pregnancy Category C)

Dapsone is an antibiotic belonging to the class of sulphones recommended for the treatment of leprosy. Owing to its antinflammatory properties, it is widely used by dermatologists in different skin diseases, including AIBDs (Kushner et al., 2018). It is a safe option in pregnant patients without glucose-6-phosphate dehydrogenase (G6PDH) deficiency and can be used during lactation monitoring the baby for hemolysis and G6PDH deficiency (Kushner et al., 2018). Data on its use for treating pemphigus during pregnancy are limited. To our knowledge, there is only one report in the literature describing the use of dapsone for treating PV during pregnancy, leading to disease control. However, in the same report, a stillborn likely not related to dapsone use was recorded (Terpstra et al., 1979). Dapsone as monotherapy (Tani et al., 2015) or in combination with systemic corticosteroids (Kirtschig et al., 1994; Soares et al., 2018) may be a good option in patients with PG showing a tendency to persist after delivery.

### Rituximab (FDA Pregnancy Category C)

Rituximab is a chimeric, humanized anti-CD20 monoclonal antibody exerting its effects in pemphigus through depletion of B cells, which are responsible for the production of anti-desmoglein autoantibodies.

Although current evidence indicates no increased rate of congenital malformations (Ojeda-Uribe et al., 2013; Sangle et al., 2013), rituximab may cross the transplacental barriers, particularly in the third trimester, and its use in fertile women should take into consideration the risk of fetal lymphocyte B depletion (Klink et al., 2008; Chakravarty et al., 2011). Furthermore, it is possible that rituximab is associated with prematurity and spontaneous abortion (Chakravarty et al., 2011). For these reasons, women are advised to try to conceive at least 12 months after the last rituximab administration (Kushner et al., 2018). However, recent reports (Lake et al., 2017; Vassallo et al., 2017) emphasized that clinical remission of pemphigus may be achieved using rituximab in the pre-conception period, allowing to discontinue immunosuppressive drugs contraindicated in pregnancy. Interestingly, rituximab has been successfully used either after delivery for treating a recalcitrant PG case (Cianchini et al., 2007) and during pregnancy for preventing PG relapse (Tourte et al., 2017).

### Intravenous Immunoglobulin Therapy (FDA Pregnancy Category C)

IVIg is a therapeutic option used for treating pemphigus patients with inadequate response or intolerance to systemic corticosteroids or conventional immunosuppressants (Bystryn et al., 2002; Amagai et al., 2009). IVIg acts by blocking the Fc receptor on the surface of macrophages and inhibiting the autoantibody production (Tavakolpour et al., 2017). Its safety during pregnancy has been demonstrated also by studies conducted for a variety of antibody-mediated autoimmune diseases other than pemphigus (Perricone et al., 2008; Sun et al., 2016). In addition, IVIg does not increase the risk of infection and seem to reduce the risk of neonatal pemphigus (Ahmed and Gürcan, 2011). As proof of this, Ono et al. (2018) showed in an experimental study on mouse model that IVIg prevented the transplacental maternal-to-fetal autoantibody transfer. Despite the above-mentioned advantages, the high cost of IVIg relegate it to second-line treatment in pregnant patients with corticosteroid-unresponsive pemphigus (Tavakolpour et al., 2017). Bostan et al. (2020) recently reported on a patient with PV begun during pregnancy who was successfully treated with a combination of IVIg and systemic corticosteroids as first-line option. The most relevant study on this topic was published in 2011 by Ahmed and Gürcan (2011), who analyzed the outcomes of eight patients with active PV during pregnancy treated with IVIg at a dosage of 2 g/kg/cycle. They found that IVIg can be useful and safe in treating pregnant patients with PV also in early phases of pregnancy. In turn, IVIg represents a good option for PG during the gestational period either as monotherapy (Nguyen et al., 2015) or combined with systemic corticosteroids (Doiron and Pratt, 2010; De la Calle et al., 2017).

### Plasmapheresis/Plasma Exchange

Plasmapheresis is a procedure consisting in removing plasma, which undergoes further processing to eliminate pathological substances such as autoantibodies, and then is returned (Reeves and Winters, 2014). In plasma exchange, the removed plasma is discarded and replaced with either a colloid solution or a combination of crystalloid and colloid solutions (Reeves and Winters, 2014). These procedures are used as an alternative therapeutic option for severe or refractory pemphigus (Nagasaka et al., 2008). Plasmapheresis and plasma exchange are considered to be a safe procedure during pregnancy, and a number of authors reported on cases of PV successfully treated with plasmapheresis/plasma exchange during pregnancy (Piontek et al., 2000; Shieh et al., 2004; Gushi et al., 2008). Plasmapheresis/plasma exchange has been performed either in combination with systemic corticosteroids (Patsatsi et al., 2012) or as monotherapy also for PG (Van de Wiel et al., 1980).

### Tumour Necrosis Factor α (TNF-α) Inhibitors

Although TNF-α has been suggested to play a role in inducing acantholysis in pemphigus, results on the effectiveness of TNF-α inhibitors, such as etanercept and infliximab, in AIBDs are conflicting (Jacobi et al., 2005; Fiorentino et al., 2011; Hall et al., 2015). However, the potential use of these drugs during pregnancy and mostly during breastfeeding needs always to be considered. A systematic review on pregnant patients with inflammatory bowel diseases revealed that the rate of adverse pregnancy outcome and malformation in patients treated with TNF-α antagonists is not significantly higher than expected in the general population (Nielsen et al., 2013). However, possible increased risk of neonatal infections has been suggested if these drugs are administered during the third trimester (Ostensen, 2014). The EULAR task force recommended continuation of TNF-α antagonists during the first part of pregnancy and suggested etanercept and certolizumab for use throughout pregnancy due to low rate of transplacental passage (Götestam Skorpen et al., 2016). No data are available for the treatment of PG with TNFα inhibitors to date.

## Treatment of Autoimmune Bullous Diseases During Lactation

Topical corticosteroids and topical calcineurin inhibitors are admitted during lactation unless they are applied in the nipple area before breastfeeding (Butler et al., 2014). In the same way, systemic corticosteroids are considered to be compatible with lactation, with the *caveat* that they should be administered at least 4 h before breastfeeding (Temprano et al., 2005). No active metabolites of mercaptopurine were detected in the blood of breastfed sons of mothers in treatment with azathioprine and no azathioprine-associated adverse effects have been observed (Angelberger et al., 2011). For these reasons, the American College of Rheumatology declared that azathioprine is conditionally recommended as compatible with breastfeeding (Birru Talabi and Clowse, 2020). However, caution should be exercised in thiopurine methyltransferase-deficient individuals (Götestam Skorpen et al., 2016) .While controlled trials are lacking, it is conceivable that IVIg is safe to be used during breastfeeding (Achiron et al., 2004). In a recent literature review, Götestam Skorpen et al. reported no adverse events in breastfed children of mothers on IVIg in most cases (n=146/149) and a transient rash only in a child (Götestam Skorpen et al., 2016). For these reasons, they are considered compatible with breastfeeding. (Götestam Skorpen et al., 2016) Plasmapheresis and immunoadsorption have been shown to be safe and well-tolerated in breastfeeding (Dittrich et al., 2002). Although rituximab has been recommended to be avoided during breastfeeding due to lack of data (Götestam Skorpen et al., 2016), it may be regarded as compatible with breastfeeding if no other options are available, since it is excreted in minimal amounts into human breast milk, likely owing to its large molecular size that impedes the transfer across the mammary tissues (Bragnes et al., 2017). TNFα antagonists, which are either minimally excreted in breast milk or scarcely absorbed, have been considered compatible with breastfeeding by the EULAR task force (Götestam Skorpen et al., 2016). Conversely, methotrexate, cyclophosphamide and mycophenolate are considered not compatible with lactation by the American College of Rheumatology and the British Society of Rheumatology due to the high risk of being transferred into breast milk (Birru Talabi and Clowse, 2020).

## Conclusion

The role of pre-pregnancy counseling is of primary importance in patients affected by pemphigus who plan to become pregnant. The most relevant aim is to induce and maintain a stable disease remission. The minimum therapeutically effective dose should be administered during pregnancy in order to prevent disease flares, which are linked to adverse pregnancy outcomes. The treatment of both pemphigus and PG mainly relies on systemic corticosteroids. However, patients who are refractory or intolerant to these agents may be treated with steroid-sparing treatments, such as dapsone, IVIg, or plasmapheresis.

## Author Contributions

GG and AM designed and wrote the initial draft of the manuscript. FD and EB reviewed the paper and provided critical intellectual input. All authors contributed to the article and approved the submitted version.

## Conflict of Interest

The authors declare that the research was conducted in the absence of any commercial or financial relationships that could be construed as a potential conflict of interest.

## References

[B1] Achiron A. Kishner I. Dolev M. Stern Y. Dulitzky M. Schiff E. (2004). Effect of intravenous immunoglobulin treatment on pregnancy and postpartum-related relapses in multiple sclerosis. J. Neurol. 251, 1133–1137. 10.1007/s00415-004-0495-z 15372259

[B2] Ahmed A. R. Gürcan H. M. (2011). Use of intravenous immunoglobulin therapy during pregnancy in patients with pemphigus vulgaris. J. Eur. Acad. Dermatol. Venereol. 25, 1073–1079. 10.1111/j.1468-3083.2010.03925.x 21143649

[B3] Akbari M. Shah S. Velayos F. S. Mahadevan U. Cheifetz A. S. (2013). Systematic review and meta-analysis on the effects of thiopurines on birth outcomes from female and male patients with inflammatory bowel disease. Inflammation Bowel Dis. 19, 15–22. 10.1002/ibd.22948 22434610

[B4] Amagai M. Ikeda S. Shimizu H. Iizuka H. Hanada K. Aiba S. (2009). A randomized double-blind trial of intravenous immunoglobulin for pemphigus. J. Am. Acad. Dermatol. 60, 595–603. 10.1016/j.jaad.2008.09.052 19293008

[B5] Amber K. T. Murrell D. F. Schmidt E. Joly P. Borradori L. (2018). Autoimmune Subepidermal Bullous Diseases of the Skin and Mucosae: Clinical Features, Diagnosis, and Management. Clin. Rev. Allergy Immunol. 54, 26–51. 10.1007/s12016-017-8633-4 28779299

[B6] Ambros-Rudolph C. M. Müllegger R. R. Vaughan-Jones S. A. Kerl H. Black M. M. (2006). The specific dermatoses of pregnancy revisited and reclassified: results of a retrospective two-center study on 505 pregnant patients. J. Am. Acad. Dermatol. 54, 395–404. 10.1016/j.jaad.2005.12.012 16488288

[B7] Angelberger S. Reinisch W. Messerschmidt A. Miehsler W. Novacek G. Vogelsang H. (2011). Long-term follow-up of babies exposed to azathioprine in utero and via breastfeeding. J. Crohns Colitis 5, 95–100. 10.1016/j.crohns.2010.10.005 21453877

[B8] Beard M. P. Millington G. W. (2012). Recent developments in the specific dermatoses of pregnancy. Clin. Exp. Dermatol. 37, 1–5. 10.1111/j.1365-2230.2011.04173.x 22007708

[B9] Birru Talabi M. Clowse M. E. B. (2020). Antirheumatic medications in pregnancy and breastfeeding. Curr. Opin. Rheumatol. 32, 238–246. 10.1097/BOR.0000000000000710 32205567

[B10] Bostan E. Gülseren D. Ersoy Evans S. Elçin G. Karaduman A. Atakan N. (2020). Efficacious treatment of pemphigus vulgaris by intravenous immunoglobulin during pregnancy and postpartum period. Dermatol. Ther. 33, e13187. 10.1111/dth.13187 31830346

[B11] Bragnes Y. Boshuizen R. de Vries A. Lexberg Å. Østensen M. (2017). Low level of Rituximab in human breast milk in a patient treated during lactation. Rheumatol. (Oxford) 56, 1047–1048. 10.1093/rheumatology/kex039 28339781

[B12] Butler D. C. Heller M. M. Murase J. E. (2014). Safety of dermatologic medications in pregnancy and lactation: Part II. Lactation. J. Am. Acad. Dermatol. 70, 417.e1–417427. 10.1016/j.jaad.2013.09.009 24528912

[B13] Bystryn J. C. Jiao D. Natow S. (2002). Treatment of pemphigus with intravenous immunoglobulin. J. Am. Acad. Dermatol. 47, 358–363. 10.1067/mjd.2002.122735 12196744

[B14] Çayırlı M. Tunca M. Akar A. Akpak Y. K. (2015). Favourable outcome of pregnancy in a patient with pemphigus vulgaris. J. Obstet. Gynaecol. 35, 747–748. 10.3109/01443615.2014.993939 25546519

[B15] Chakravarty E. F. Murray E. R. Kelman A. Farmer P. (2011). Pregnancy outcomes after maternal exposure to rituximab. Blood 117 (5), 1499–1506. 10.1182/blood-2010-07-295444 21098742

[B16] Chi C. C. Wang S. H. Kirtschig G. (2016). Safety of Topical Corticosteroids in Pregnancy. JAMA Dermatol. 152, 934–935. 10.1001/jamadermatol.2016.1009 27366995

[B17] Chowdhury M. M. Natarajan S. (1998). Neonatal pemphigus vulgaris associated with mild oral pemphigus vulgaris in the mother during pregnancy. Br. J. Dermatol. 139, 500–503. 10.1046/j.1365-2133.1998.02418.x 9767299

[B18] Cianchini G. Masini C. Lupi F. Corona R. De Pità O. Puddu P. (2007). Severe persistent pemphigoid gestationis: long-term remission with rituximab. Br. J. Dermatol. 157, 388–389. 10.1111/j.1365-2133.2007.07982.x 17553047

[B19] Committee on Obstetric Practice Society for Maternal-Fetal Medicine (2019). Immune modulating therapies in pregnancy and lactation. ACOG Committee Opinion No. 776. American College of Obstetricians and Gynecologists. Obstet. Gynecol. 133, e287–e295. 10.1097/AOG.0000000000003176 30913201

[B20] Cozzani E. Basso M. Parodi A. Rebora A. (2005). Pemphigoid gestationis post partum after changing husband. Int. J. Dermatol. 44, 1057–1058. 10.1111/j.1365-4632.2004.02548.x 16409278

[B21] Daneshpazhooh M. Chams-Davatchi C. Valikhani M. Aghabagheri A. Mortazavizadeh S. M. Barzegari M. (2011). Pemphigus and pregnancy: a 23-year experience. Indian J. Dermatol. Venereol. Leprol. 77, 534. 10.4103/0378-6323.82404 21727712

[B22] De la Calle M. Vidaurrázaga C. Martinez N. González-Beato M. Antolín E. Bartha J. L. (2017). Successful treatment of a severe early onset case of pemphigoid gestationis with intravenous immunoglobulin in a twin pregnancy conceived with in vitro fertilisation in a primigravida. J. Obstet. Gynaecol. 37, 246–247. 10.1080/01443615.2016.1244809 27922278

[B23] Dittrich E. Schmaldienst S. Langer M. Jansen M. Hörl W. H. Derfler K. (2002). Immunoadsorption and plasma exchange in pregnancy. Kidney Blood Press Res. 25, 232–239. 10.1159/000066343 12424425

[B24] Djahansouzi S. Nestle-Kraemling C. Dall P. Bender H. G. Hanstein B. (2003). Herpes gestationis may present itself as a paraneoplastic syndrome of choriocarcinoma-a case report. Gynecol. Oncol. 89, 334–337. 10.1016/s0090-8258(03)00070-2 12714000

[B25] do Valle Chiossi M. P. Costa R. S. Ferreira Roselino A. M. (2000). Titration of herpes gestationis factor fixing to C3 in pemphigoid herpes gestationis associated with choriocarcinoma. Arch. Dermatol. 136, 129–130. 10.1001/archderm.136.1.129-a 10632227

[B26] Doiron P. Pratt M. (2010). Antepartum intravenous immunoglobulin therapy in refractory pemphigoid gestationis: case report and literature review. J. Cutan. Med. Surg. 14, 189–192. 10.2310/7750.2009.09001 20642990

[B27] Egami S. Yamagami J. Amagai M. (2020). Autoimmune bullous skin diseases, pemphigus and pemphigoid. J. Allergy Clin. Immunol. 145, 1031–1047. 10.1016/j.jaci.2020.02.013 32272980

[B28] Feliciani C. Genovese G. D’Astolto R. Pontini P. Marzano A. V. (2019). Autoimmune bullous diseases during pregnancy: insight into pathogenetic mechanisms and clinical features. G. Ital. Dermatol. Venereol. 154, 256–262. 10.23736/S0392-0488.18.06153-9 30375213

[B29] Fiorentino D. F. Garcia M. S. Rehmus W. Kimball A. B. (2011). A pilot study of etanercept treatment for pemphigus vulgaris. Arch. Dermatol. 147, 117–118. 10.1001/archdermatol.2010.409 21242406

[B30] Flint J. Panchal S. Hurrell A. van de Venne M. Gayed M. Schreiber K. (2016). BSR and BHPR guideline on prescribing drugs in pregnancy and breastfeeding-Part I: standard and biologic disease modifying anti-rheumatic drugs and corticosteroids. Rheumatol. (Oxford) 55, 1693–1697. 10.1093/rheumatology/kev404 26750124

[B31] Fukuda S. Ishii N. Hamada T. Ohyama B. Momosaki N. Karashima T. (2012). A case of herpes gestationis: follow-up study of autoantibodies using enzyme-linked immunosorbent assay and immunoblotting. Indian J. Dermatol. Venereol. Leprol. 78, 199–201. 10.4103/0378-6323.93646 22421659

[B32] Goldstein L. H. Dolinsky G. Greenberg R. Schaefer C. Cohen-Kerem R. Diav-Citrin O. (2007). Pregnancy outcome of women exposed to azathioprine during pregnancy. Birth Defects Res. A. Clin. Mol. Teratol. 79, 696–701. 10.1002/bdra.20399 17847119

[B33] Götestam Skorpen C. Hoeltzenbein M. Tincani A. Fischer-Betz R. Elefant E. Chambers C. (2016). The EULAR points to consider for use of antirheumatic drugs before pregnancy, and during pregnancy and lactation. Ann. Rheumatol. Dis. 75, 795–810. 10.1136/annrheumdis-2015-208840 26888948

[B34] Green D. Maize J. C. (1982). Maternal pemphigus vulgaris with in vivo bound antibodies in the stillborn fetus. J. Am. Acad. Dermatol. 7, 388–392. 10.1016/s0190-9622(82)70125-2 6890077

[B35] Gushi M. Yamamoto Y. Mine Y. Awazawa R. Nonaka K. Taira K. (2008). Neonatal pemphigus vulgaris. J. Dermatol. 35, 529–535. 10.1111/j.1346-8138.2008.00515.x 18789074

[B36] Hall R. P. Fairley J. Woodley D. Werth V. P. Hannah D. Streilein R. D. (2015). A multicentre randomized trial of the treatment of patients with pemphigus vulgaris with infliximab and prednisone compared with prednisone alone. Br. J. Dermatol. 172, 760–768. 10.1111/bjd.13350 25123295 PMC4326612

[B37] Hern S. Vaughan Jones S. A. Setterfield J. Du Peloux Menag H. Greaves M. W. Rowlatt R. (1998). Pemphigus vulgaris in pregnancy with favourable foetal prognosis. Clin. Exp. Dermatol. 23, 260–263. 10.1046/j.1365-2230.1998.00370.x 10233621

[B38] Hone N. L. Mutasim D. F. (2016). Pemphigus vulgaris successfully treated with doxycycline monotherapy. Cutis 97, E25–E27. 27416094

[B39] Ingen-Housz-Oro S. Sbidian E. Ortonne N. Poirier E. Chosidow O. Wolkenstein P. (2016). Pemphigoid gestationis revealing a denial of pregnancy. J. Eur. Acad. Dermatol. Venereol. 30, 1411–1413. 10.1111/jdv.13257 26299950

[B40] Jacobi A. Shuler G. Hertl M. (2005). Rapid control of therapy-refractory pemphigus vulgaris by treatment with the tumour necrosis factor-alpha inhibitor infliximab. Br. J. Dermatol. 153, 448–449. 10.1111/j.1365-2133.2005.06744.x 16086769

[B41] Kardos M. Levine D. Gürcan H. M. Ahmed R. A. (2009). Pemphigus vulgaris in pregnancy: analysis of current data on the management and outcomes. Obstet. Gynecol. Surv. 64, 739–749. 10.1097/OGX.0b013e3181bea089 19849866

[B42] Kirtschig G. Collier P. M. Emmerson R. W. Wojnarowska F. (1994). Severe case of pemphigoid gestationis with unusual target antigen. Br. J. Dermatol. 131, 108–111. 10.1111/j.1365-2133.1994.tb08466.x 8043401

[B43] Klink D. T. van Elburg R. M. Schreurs M. W. van Well G. T. (2008). Rituximab administration in third trimester of pregnancy suppresses neonatal B-cell development. Clin. Dev. Immunol. 2008, 271363. 10.1155/2008/271363 18596903 PMC2438602

[B44] Kokolios M. Lamprou F. Stylianidou D. Sotiriadis D. Patsatsi A. (2017). New onset pemphigus foliaceus during pregnancy: A rare case. Int. J. Womens. Dermatol. 4, 109–112. 10.1016/j.ijwd.2017.10.010 29872686 PMC5986257

[B45] Kridin K. (2018). Pemphigus group: overview, epidemiology, mortality, and comorbidities. Immunol. Res. 66, 255–270. 10.1007/s12026-018-8986-7 29479654

[B46] Kushner C. J. Concha J. S. S. Werth V. P. (2018). Treatment of Autoimmune Bullous Disorders in Pregnancy. Am. J. Clin. Dermatol. 19, 391–403. 10.1007/s40257-018-0342-0 29392620

[B47] Lake E. P. Huang Y. H. Aronson I. K. (2017). Rituximab treatment of pemphigus in women of childbearing age: experience with two patients. J. Dermatol. Treat. 28, 751–752. 10.1080/09546634.2016.1255302 27796136

[B48] Lin L. Zeng X. Chen Q. (2015). Pemphigus and pregnancy. Analysis and summary of case reports over 49 years. Saudi Med. J. 36, 1033–1038. 10.15537/smj.2015.9.12270 26318458 PMC4613625

[B49] Moncada B. Kettelsen S. Hernández-Moctezuma J. L. Ramirez F. (1982). Neonatal pemphigus vulgaris: role of passively transferred pemphigus antibodies. Br. J. Dermatol. 106, 465–467. 10.1111/j.1365-2133.1982.tb04542.x 7073971

[B50] Murase J. E. Heller M. M. Butler D. C. (2014). Safety of dermatologic medications in pregnancy and lactation: Part I. Pregnancy J. Am. Acad. Dermatol. 70, 401.e1–401415. 10.1016/j.jaad.2013.09.010 24528911

[B51] Nagasaka T. Fujii Y. Ishida A. Handa M. Tanikawa A. Amagai M. (2008). Evaluating efficacy of plasmapheresis for patients with pemphigus using desmoglein enzyme-linked immunosorbent assay. Br. J. Dermatol. 158 (4), 685–690. 10.1111/j.1365-2133.2007.08416.x 18241273

[B52] Nguyen T. Alraqum E. Razzaque Ahmed A. (2015). Positive clinical outcome with IVIg as monotherapy in recurrent pemphigoid gestationis. Int. Immunopharmacol. 26, 1–3. 10.1016/j.intimp.2015.02.038 25765353

[B53] Nielsen O. H. Loftus E. V. Jr Jess T. (2013). Safety of TNF-alpha inhibitors during IBD pregnancy: a systematic review. BMC Med. 11, 174. 10.1186/1741-7015-11-174 23902720 PMC3734216

[B54] Nørgård B. Pedersen L. Fonager K. Rasmussen S. N. Sørensen H. T. (2003). Azathioprine, mercaptopurine and birth outcome: a population-based cohort study. Aliment Pharmacol. Ther. 17, 827–834. 10.1046/j.1365-2036.2003.01537.x 12641505

[B55] Ojeda-Uribe M. Afif N. Dahan E. Sparsa L. Haby C. Sibilia J. (2013). Exposure to abatacept or rituximab in the first trimester of pregnancy in three women with autoimmune diseases. Clin. Rheumatol. 32, 695–700. 10.1007/s10067-012-2156-4 23292481

[B56] Ono S. Egawa G. Honda T. Kabashima K. (2018). Intravenous immunoglobulin treatment abrogates transplacental autoantibody transfer in a murine pemphigus model. J. Allergy Clin. Immunol. 141 (6), 2273–2276.e1. 10.1016/j.jaci.2017.12.985 29360528

[B57] Ostensen M. (2014). Safety issues of biologics in pregnant patients with rheumatic diseases. Ann. N. Y. Acad. Sci. 1317, 32–38. 10.1111/nyas.12456 24840548

[B58] Patsatsi A. Vavilis D. Tsikeloudi M. Kalabalikis D. Sotiriadis D. (2012). Refractory pemphigoid gestationis postpartum. Acta Obstet. Gynecol. Scand. 91, 636–637. 10.1111/j.1600-0412.2012.01379.x 22360419

[B59] Patsatsi A. Marinovic B. Murrell D. (2019). Autoimmune bullous diseases during pregnancy: Solving common and uncommon issues. Int. J. Womens Dermatol. 5, 166–170. 10.1016/j.ijwd.2019.01.003 31360750 PMC6637227

[B60] Perez-Garcia L. F. Dolhain R. J. E. M. Vorstenbosch S. Bramer W. van Puijenbroek E. Hazes J. M. W. (2020). The effect of paternal exposure to immunosuppressive drugs on sexual function, reproductive hormones, fertility, pregnancy and offspring outcomes: a systematic review. Hum. Reprod. 10.1093/humupd/dmaa022 PMC7600290 32743663

[B61] Perricone R. De Carolis C. Kröegler B. Greco E. Giacomelli R. Cipriani P. (2008). Intravenous immunoglobulin therapy in pregnant patients affected with systemic lupus erythematosus and recurrent spontaneous abortion. Rheumatol. (Oxford) 47, 646–651. 10.1093/rheumatology/ken046 18346976

[B62] Piontek J. O. Borberg H. Sollberg S. Krieg T. Hunzelmann N. (2000). Severe exacerbation of pemphigus vulgaris in pregnancy: successful treatment with plasma exchange. Br. J. Dermatol. 143, 455–456. 10.1046/j.1365-2133.2000.03686.x 10951169

[B63] Piva I. Lo Monte G. Graziano A. Marci R. (2014). Herpes Gestationis after Ovodonation: Is Placenta the only Target of the Immune Reaction? J. Clin. Diagn. Res. 8, OD01–OOD2. 10.7860/JCDR/2014/8727.5103 PMC4290294 25584273

[B64] Reeves H. M. Winters J. L. (2014). The mechanisms of action of plasma exchange. Br. J. Haematol. 164 (3), 342–351. 10.1111/bjh.12629 24172059

[B65] Sammaritano L. R. Bermas B. L. Chakravarty E. E. Chambers C. Clowse M. E. B. Lockshin M. D. (2020). 2020 American College of Rheumatology Guideline for the Management of Reproductive Health in Rheumatic and Musculoskeletal Diseases. Arthritis Rheumatol. 72, 529–556. 10.1002/art.41191 32090480

[B66] Sangle S. R. Lutalo P. M. Davies R. J. Khamashta M. A. D’Cruz D. P. (2013). B-cell depletion therapy and pregnancy outcome in severe, refractory systemic autoimmune diseases. J. Autoimmun. 43, 55–59. 10.1016/j.jaut.2013.03.001 23608146

[B67] Shieh S. Fang Y. V. Becker J. L. Holm A. Beutner E. H. Helm T. N. (2004). Pemphigus, pregnancy, and plasmapheresis. Cutis 73, 327–329. 15186047

[B68] Slazinski L. Degefu S. (1982). Herpes gestationis associated with choriocarcinoma. Arch. Dermatol. 118, 425–428. 10.1001/archderm.1982.01650180059019 7046639

[B69] Soares K. S. Lehmann P. M. Hofmann S. C. (2018). Pemphigoid gestationis with lethal fetal malformation and postpartum persistence. J. Dtsch. Dermatol. Ges. 16, 775–777. 10.1111/ddg.13540 29873913

[B70] Sun D. Shehata N. Ye X. Y. Gregorovich S. De France B. Arnold D. M. (2016). Corticosteroids compared with intravenous immunoglobulin for the treatment of immune thrombocytopenia in pregnancy. Blood 128, 1329–1335. 10.1182/blood-2016-04-710285 27402971

[B71] Tani N. Kimura Y. Koga H. Kawakami T. Ohata C. Ishii N. (2015). Clinical and immunological profiles of 25 patients with pemphigoid gestationis. Br. J. Dermatol. 172, 120–129. 10.1111/bjd.13374 25154546

[B72] Tavakolpour S. Mirsafaei H. S. Delshad S. (2017). Management of pemphigus disease in pregnancy. Am. J. Reprod. Immunol. 77. 10.1111/aji.12601 27862562

[B73] Temprano K. K. Bandlamudi R. Moore T. L. (2005). Antirheumatic drugs in pregnancy and lactation. Semin. Arthritis Rheumatol. 35, 112–121. 10.1016/j.semarthrit.2005.05.002 16194696

[B74] Terpstra H. de Jong M. C. Klokke A. H. (1979). In vivo bound pemphigus antibodies in a stillborn infant. Passive intrauterine transfer of pemphigus vulgaris? Arch. Dermatol. 115, 316–319. 373633

[B75] Tourte M. Brunet-Possenti F. Mignot S. Gavard L. Descamps V. (2017). Pemphigoid gestationis: a successful preventive treatment by rituximab. J. Eur. Acad. Dermatol. Venereol. 31, e206–e207. 10.1111/jdv.13962 27606493

[B76] Van de Wiel A. Hart H. C. Flinterman J. Kerckhaert J. A. Du Boeuff J. A. Imhof J. W. (1980). Plasma exchange in herpes gestationis. Br. Med. J. 281, 1041–1042. 10.1136/bmj.281.6247.1041-a PMC1714448 7000286

[B77] Vassallo C. Grassi S. Tagliabue E. Piccolo A. Brazzelli V. (2017). Pregnancy outcome after rituximab treatment before conception in patients affected by severe pemphigus vulgaris/superficialis. J. Eur. Acad. Dermatol. Venereol. 31, e331–e333. 10.1111/jdv.14119 28079925

[B78] Veiga V. F. Santos F. Antunes A. Duarte I. (2018). Pemphigoid gestationis. BMJ Case Rep., bcr2018225242. 10.1136/bcr-2018-225242 PMC6011501 29914905

[B79] Zhao C. Y. Murrell D. F. (2015). Autoimmune blistering diseases in females: a review. Int. J. Womens Dermatol. 1, 4–12. 10.1016/j.ijwd.2015.01.002 28491949 PMC5418673

[B80] Zhao C. Y. Chiang Y. Z. Murrell D. F. (2016). Neonatal Autoimmune Blistering Disease: A Systematic Review. Pediatr. Dermatol. 33, 367–374. 10.1111/pde.12859 27086740

